# Bilateral hand-assisted laparoscopic nephrectomy in adult polycystic kidney disease patients: a UK centre experience

**Published:** 2012-06-18

**Authors:** C Mak DK, CP Ilie, A Abedin, L Gommersall, C Luscombe, A Golash

**Affiliations:** * Department of Urology, “Carol Davila” University of Medicine and Pharmacy, Bucharest, Romania; ** Department of Urology, University Hospital of North Staffordshire, United Kingdom

**Keywords:** laparoscopy, nephrectomy, laparoscopic nephrectomy, hand-assisted laparoscopic nephrectomy, adult polycystic kidney disease

## Abstract

**Purpose:** We report our experience with bilateral hand-assisted laparoscopic nephrectomy in patients with adult polycystic kidney disease.

**Materials & methods:** Between November 2009 and November 2010, 3 patients with adult polycystic kidney disease underwent bilateral hand-assisted laparoscopic nephrectomy in our institution. Indications for bilateral nephrectomy included recurrent cyst hemorrhage, impaired gastrointestinal function and early satiety due to direct intestinal compression by large polycystic kidneys, and anatomical lack of space for future renal transplantation. We retrospectively reviewed the records of these patients and we are reporting our experience.

**Results:** All three patients successfully underwent bilateral hand-assisted laparoscopic nephrectomy with a mean operating time of 208 minutes (range 195 to 220). There were no conversions to open procedure. Blood loss was less than 100 ml in all cases. Mean renal unit size was of 2037 g (range 1798 to 2214). Hospital stay ranged from 10 to 12 days. One patient developed a chest infection postoperatively and suffered from a prolonged ileus. Another patient developed a retroperitoneal hematoma, which was treated conservatively.

**Conclusions:** Bilateral hand-assisted laparoscopic nephrectomy is a feasible and safe procedure in adult polycystic kidney disease patients, which has potential benefits of a shorter hospital stay and reduced morbidity and mortality in comparison to open procedure.

## Introduction

Adult polycystic kidney disease (APCKD) is an autosomal dominant genetic disorder that is characterized by the development of renal cysts and other extra-renal manifestations, including cysts in other organs, intracranial aneurysms, and mitral valve prolapse. It has a prevalence of 0.1 to 0.25% [**1**]. APCKD is responsible for causing 10% of all end-stage renal failure (ESRF). In such patients, renal cysts can enlarge significantly and cause symptoms including hypertension, abdominal pain, infection, early satiety, and haematuria. In addition, significantly enlarged cysts can impinge on anatomical space required for future renal transplantation. Minimally invasive techniques, such as percutaneous cyst aspiration and laparoscopic cyst decortication, can provide symptomatic relief in some patients [**2**]. For patients with enlarged symptomatic polycystic kidneys and ESRF, who have failed conservative management, unilateral or bilateral nephrectomy is an option.

Open nephrectomy in APCKD has been associated with significant rates of morbidity (12%) and mortality (5%) [**3**]. Subsequently, the frequency of open nephrectomy in APCKD patients decreased in the 1980s. With the evolution of laparoscopic techniques, reports on the feasibility of laparoscopic nephrectomy in symptomatic APCKD patients have been increasingly described. Since Elashry et al. reported the first unilateral laparoscopic nephrectomy for APCKD in 1996 [**4**], synchronous bilateral laparoscopic nephrectomy has been described. This is an uncommon procedure and presents unique challenges to the surgeon.

We describe our experience of the surgical management of symptomatic patients with APCKD and ESRD, using a hand-assisted laparoscopic (HAL) technique, at a single UK tertiary referral university hospital.

## Materials and methods

We retrospectively reviewed the records of 3 patients, with symptomatic APCKD, who underwent bilateral HAL nephrectomy, from November 2009 to November 2010. Indications for bilateral nephrectomy included recurrent cyst hemorrhage, impaired gastrointestinal function and early satiety due to direct intestinal compression by the large polycystic kidneys, and anatomical lack of space for future renal transplantation. All patients were established on haemodialysis and awaiting renal transplantation.

**Fig. 1:**
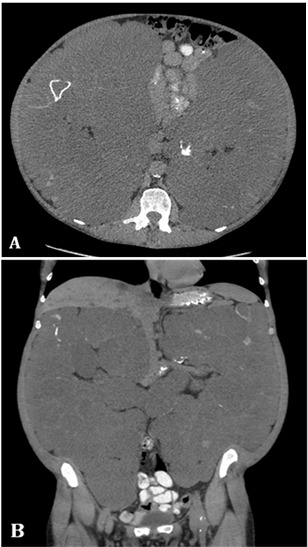
Pre-operative CT scan demonstrating significantly enlarged polycystic kidneys removed using hand-assisted laparoscopic techniques. A. axial view. B. coronal view

**Fig. 2:**
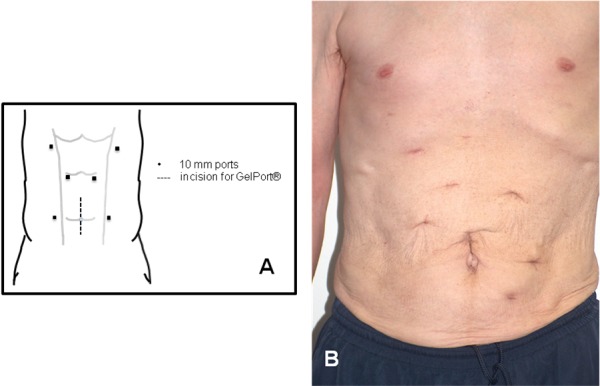
A. Port placement for bilateral HAL nephrectomy. B. Photo of patient demonstrating port site locations post-operatively

Prior to surgery, patients underwent computerized tomography of the native kidneys to determine renal vessel anatomy and renal size (**Figure 1**). The following surgical technique was employed. After inducing general anesthesia, the patient was put in a right-sided semi-lateral position with a view to performing a left nephrectomy initially. The patient was secured by using adhesive tape and a beanbag bolster. A 10-12 cm vertical midline incision was made incorporating the umbilicus to allow for GelPort® (Applied Medical, USA) placement. Insufflation was performed through a 10 mm blunt port placed through the GelPort® device. To prevent trauma to abdominal viscera, the abdomen was visualised through a 10mm 30° lens whilst three 10 mm ports were placed under direct vision. The placement of the working ports is shown in **Figure 2**. The descending colon was mobilized and access to the retroperitoneum was achieved. With the colon reflected medially, the renal hilum was identified and carefully dissected. Hem-o-lok clips were applied to both the renal artery and renal vein separately before the vessels were divided. The ureter was located and divided with the LigaSure™ device (Covidien, USA). Using gentle manual retraction, the left kidney was then carefully dissected and all small vessels were controlled with diathermy and LigaSure™. The specimen was extracted through the hand port. Cyst decortication was performed extracorporeally as they were exposed to decrease the overall kidney size and aid extraction of the specimen. A drain was inserted to the left renal bed through one of the 10 mm port sites and sutured into place with a 1-0 silk suture. The skin of the remaining 10 mm port sites were closed with surgical adhesive. The patient was then repositioned in a left-sided flank position for the subsequent right HAL nephrectomy. Three 10 mm ports were inserted under direct vision (**Figure 1**)). The liver was retracted with a laparoscopic Babcock clamp inserted into the xiphoid port and attached lateral to the abdominal wall. The ascending colon and hepatic flexure were mobilized. The right kidney was dissected and extracted in a similar fashion as described before. A second drain was placed into the right renal bed via one of the 10 mm ports. Following removal of the GelPort®, the extraction site was closed using 3-0 polydioxanone suture. The skin was closed with a subcuticular Monocryl suture. The skin of the 10 mm port sites were closed using surgical adhesive.

For each of the treatment groups, 95% confidence intervals were calculated for the treatment’s success rates. The statistical comparison of two independent percentages was done by means of the Fisher’s exact test (2-sided, p = 0.05). If the resulting p value was < 0.05, the difference in the sample percentages was considered statistically significant.

## Results 

**Fig. 3:**
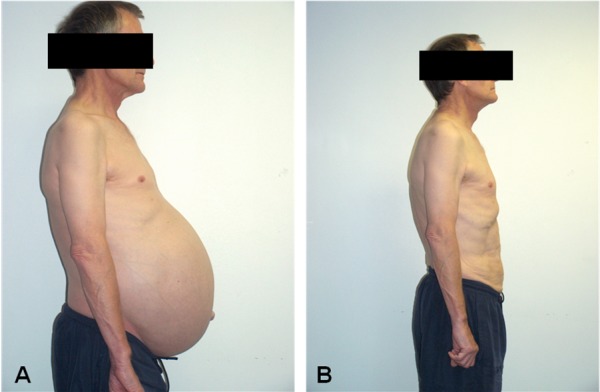
Photos illustrating patient appearances before (A) and after (B) bilateral HAL nephrectomy

The ages of patients were 36, 53, and 56 years. All the patients were male. Operating times were 195, 210 and 220 minutes. Blood loss was less than 100 ml in all 3 cases. The lengths of hospital stay were 10, 10 and 12 days. Pathological evidence of the specimens demonstrated volumes of 2321, 2420, 2876, 3206, 3342 and 4224 cm³ with a weight of 1798, 1884, 1967, 2164, 2197 and 2214 g after partial cyst decortication. There was no evidence of malignancy. **Figure 3** illustrates pre-operative and post-operative patient appearances.

All procedures were successfully performed laparoscopically. No patient required intraoperative conversion to open surgery. There were no intraoperative complications. One patient developed a chest infection, four days postoperatively, which was successfully treated with antibiotics. The same patient also developed a post-operative ileus that was resolved with conservative management. After a drop in hemoglobin levels postoperatively, one patient was found to have a left sided retroperitoneal hematoma, evident on computed tomography (CT). This patient was never haemodynamically compromised but was subsequently transfused. The hematoma was successfully treated conservatively.

## Discussion

In symptomatic APCKD patients who have failed conservative management or required anatomical space for renal transplantation, surgical treatment was required. In APCKD patients with ESRF, who are on established renal replacement therapy or have a functioning renal transplant, bilateral nephrectomy is a treatment option.

Open bilateral nephrectomy was associated with significant morbidity and mortality. Yarimizu et al. reported a mortality rate of 3.6% and morbidity rate of 45% in their study of 305 patients undergoing open bilateral nephrectomy [**5**]. Advances in technology and laparoscopic techniques have allowed bilateral nephrectomy procedures to be performed with improved outcomes [**6**].

Elashry et al. first highlighted the potential use of laparoscopy in performing nephrectomy in APCKD patients [**4**]. Since then, other groups have demonstrated the safety and advantages of laparoscopic nephrectomy in APCKD patients [**7-9**]. Bales et al. [**10**] reported the first laparoscopic bilateral nephrectomy. They successfully utilized a transperitoneal approach to perform bilateral nephrectomy for renin-mediated hypertension. Further reports demonstrating the feasibility of laparoscopic bilateral nephrectomy have since been published [**11,12**]. Fornara et al. highlighted the advantages of laparoscopic bilateral nephrectomy over open bilateral nephrectomy with shorter hospital stay, decreased postoperative analgesic use, rapid time to oral intake, and more rapid convalescence [**6**]. However, the mean operative time for laparoscopic bilateral nephrectomy was longer in comparison to open bilateral nephrectomy (195 minutes and 145 minutes respectively). Gill et al. further reinforced the benefits of laparoscopic bilateral nephrectomy over open bilateral nephrectomy [**11**], demonstrating significantly shorter hospital stays, decreased opiate use postoperatively, and more rapid resumption of oral intake. Total surgical time was again noted to be significantly longer in the laparoscopic group in comparison to the open bilateral nephrectomy group.

Laparoscopic surgeons face unique challenges in APCKD patients. Kidney dissection is complicated by increased renal size, perinephric inflammation and fibrosis, and difficulty identifying and accessing the renal hilum. Such challenges have been recognized to increase complications and prolong operative times [**12,13**]. With the advent of hand-assist devices, laparoscopic nephrectomy for APCKD can be performed more practically. Nakada et al. first introduced the principles of hand-assisted laparoscopy (HAL) to perform nephrectomy for symptomatic polycystic kidneys [**14**]. Wolf et al. [**15**] reported the advantages of HAL nephrectomy over standard laparoscopic nephrectomy. By comparing HAL and standard laparoscopic nephrectomy, they found statistically significant improvements in operative times and complication rates in the HAL nephrectomy group. They concluded that HAL improved manipulative ability and sterotactile sense, which significantly decreased operative times without compromising the benefits of laparoscopic surgery.

To date, there have been eleven published reports on HAL bilateral nephrectomy in APCKD patients, all of which demonstrate its safety and impact on reducing the length of hospital stay [**16-26**] (**Table 1**). Mean operative times range from 185 to 335 minutes and reported mean estimated blood loss range from 100 to 350 mls. The operative times and blood loss reported in our study are comparable to the reported data. The length of hospital stay in our study was longer in comparison to other studies due to postoperative events delaying discharge and patients remaining in hospital for the convenience of undergoing routine haemodialysis. The majority of the reported published studies presented no intraoperative complications as we observed in our study. Only Desai et al. reported a serosal duodenal tear, which was recognized intraoperatively and repaired laparoscopically [**24**]. We reported postoperative complications of chest infection and retroperitoneal hematoma requiring blood transfusion. Such complications have been recognized in other studies and could be considered as acceptable in high risk, immunocompromised and dialyzed patients.

In our series, no patient required conversion to open surgery. Lipke et al. reported a 22.2% conversion rate [**23**]. They found that conversions tended to occur in patients with very large kidneys, which hindered access to the renal hilum. They concluded that large kidneys of >3500cm³ are less likely to be removed laparoscopically and would be best removed through an open approach. In our study, we successfully removed renal unit sizes of up to 4224cm³ even after considerable reduction by partial cyst decortication through a laparoscopic approach. The largest kidneys removed laparoscopically in the series reported by Desai et al. were of 4200g and 5042g [**24**].

**Table 1:** Summary of published data on HAL bilateral nephrectomy in APCKD patients

	No. of patients	Mean op time (mins)	Mean estimated blood loss (ml)	Mean length of stay (days)	Mean renal unit size (g)	Conversion rate (%)	Complications
Schmidlin et al. [**16**]	1	210	300	-	-	0	
Rehman et al. [**17**]	3	330	100-200	4.3	617	0	
Jenkins et al. [**18**]	4	286	338	-	1582	0	Blood transfusion ATN of allograft & pulmonary edema Retroperitoneal collection/hematoma
Pinto et al. [**19**]	1	195	160	4	-	0	
Tobias-Machado et al. [**20**]	3	190	-	3.4	-	0	Pneumothorax
Zaman et al. [**21**]	6	185	345	-	2029	0	
Whitten et al. [**22**]	10	194	203	4.7	717	0	Dialysis fistula thrombosis Postoperative respiratory depression
Lipke et al. [**23**]	18	315	350	3.4	1768	22	Incisional hernia Wound infection Prolonged ileus
Desai et al. [**24**]	12	214	164	4.2	2311	0	Serosal duodenal tear Pulmonary embolism Blood transfusion
El-Galley et al. [**25**]	26	222	175	3	-	0	Adrenal insufficiency Pulmonary embolism Wound infection Loss of AV fistula function
Patel et al. [**26**]	3	-	-	5 (median)	-	0	
Mak et al.	3	208	100	10.6	2037	0	Chest infection Prolonged ileus Retroperitoneal hematoma Blood transfusion

Few studies have directly compared staged and synchronous nephrectomy in the setting of APCKD patients. Lucas et al. compared bilateral laparoscopic nephrectomy with a staged approach, involving unilateral nephrectomy at time of transplantation followed by contralateral laparoscopic nephrectomy after recovery, in patients with APCKD [**27**]. They found shorter operative times and estimated blood loss in the unilateral group as it would be expected. However, there was no difference in the length of hospital stay between the two groups. Although synchronous bilateral nephrectomy entails longer operative time and increased blood loss, a staged approach has the significant disadvantage of requiring an additional procedure to remove the two kidneys.

Many variations in the technique of HAL bilateral nephrectomy have been described in published studies. Desai et al. highlighted the use of the GelPort® device and subsequent port placement under direct visualization, which we advocate to avoid inadvertent visceral injury [**24**]. Desai et al. also favored extracting polycystic kidneys with the cysts kept intact as much as possible. Conversely, Whitten et al. used a vacuum curettage system to morcellate and aspirate the specimen intracorporeally [**22**]. We encourage extracorporeal cyst decortication to aid extraction of the specimen for a number of reasons. Localized chemical peritonitis has been observed with the release of cyst fluid intraperitoneally and can lead to a prolonged paralytic ileus [**11**]. More importantly, there is an association of APCKD and renal cell carcinoma [**28**]. Thus, although small, there is a potential risk of malignancy seeding by intracorporeal cyst decortication or morcellation. El-Galley et al. suggested stapling the renal vessels en bloc to reduce the complications associated with bleeding and to facilitate medical dissection of the kidney [**25**]. In the present and other studies, renal vessel ligation was performed separately. We found that the access to the renal hilum in large polycystic kidneys can be difficult and en-bloc ligation with a stapler was not feasible. We therefore advocate separate renal vessel ligation in such cases.

We commend the HAL technique to perform synchronous bilateral nephrectomy in the setting of APCKD patients. Our study adds to the growing body of literature supporting the feasibility and safety of the hand-assisted laparoscopic approach in APCKD patients, allowing for shorter hospital stay and reduced morbidity compared to an open approach. Comparative multicentre studies with more numbers are required to define the optimal bilateral HAL nephrectomy technique and report on the impact on length of hospital stay, morbidity and mortality.
